# Preservation of the Myofascial Cuff During Posterior Fossa Surgery to Reduce the Rate of Pseudomeningocele Formation and Cerebrospinal Fluid Leak: A Technical Note

**DOI:** 10.7759/cureus.946

**Published:** 2016-12-28

**Authors:** Daniel R Felbaum, Kyle Mueller, Amjad Anaizi, Robert B Mason, Walter C Jean, Jean M Voyadzis

**Affiliations:** 1 Neurosurgery, Medstar Georgetown University Hospital

**Keywords:** posterior fossa, chiari decompression, pseudomeningocele, meningitis, lumbar drain, cerebellum

## Abstract

Introduction: Suboccipital craniotomy is a workhorse neurosurgical operation for approaching the posterior fossa but carries a high risk of pseudomeningocele and cerebrospinal fluid (CSF) leak. We describe our experience with a simple T-shaped fascial opening that preserves the occipital myofascial cuff as compared to traditional methods to reduce this risk.

Methods: A single institution, retrospective review of prospectively collected database was performed of patients that underwent a suboccipital craniectomy or craniotomy. Patient data was reviewed for craniotomy or craniectomy, dural graft, and/or sealant use as well as CSF complications. A pseudomeningocele was defined as a subcutaneous collection of cerebrospinal fluid palpable clinically and confirmed on imaging. A CSF leak was defined as a CSF-cutaneous fistula manifested by CSF leaking through the wound. All patients underwent regular postoperative visits of two weeks, one month, and three months.

Results: Our retrospective review identified 33 patients matching the inclusion criteria. Overall, our cohort had a 21% (7/33) rate of clinical and radiographic pseudomeningocele formation with 9% (3/33) requiring surgical revision or a separate procedure. The rate of clinical and radiographic pseudomeningocele formation in the myofascial cuff preservation technique was less than standard techniques (12% and 31%, respectively). Revision or further surgical procedures were also reduced in the myofascial cuff preservation technique vs. the standard technique (6% vs 13%).

Conclusions: Preservation of the myofascial cuff during posterior fossa surgery is a simple and adoptable technique that reduces the rate of pseudomeningocele formation and CSF leak as compared with standard techniques.

## Introduction

Suboccipital craniotomy is a workhorse neurosurgical operation for approaching the posterior fossa [1-2]. Because of its gravity-dependent location, posterior fossa approaches carry a higher risk of pseudomeningocele and cerebrospinal fluid leak (CSF) [3]. In the reported literature, with various underlying pathologies and radiographic or clinical criteria, the rate of pseudomeningocele rate can be as high as 40% [4-5].

The technique of suboccipital craniotomy typically involves a midline or hockey stick incision, a wide subperiosteal detachment of the posterior musculature from the occipital bone, and dorsal spinal elements. The potential for dead space, combined with the difficulty in reattachment of the musculoligamentous tissues to the occipital bone, creates a direct conduit for cerebrospinal fluid to reach the skin. This is compounded by the thin and typically friable dura of the posterior fossa [6-7].

We describe our experience during posterior fossa surgery with a simple technique that preserves the occipital myofascial cuff formed from the insertion of the paraspinous musculature of the occipital bone. A T-shaped fascial opening is performed during the initial dissection that allows for watertight reattachment of the paraspinal musculature to the spinal elements and occipital bone, reducing dead space and eliminating a CSF conduit to the skin. Our hypothesis is that this simple technical nuance should reduce the rate of pseudomeningocele formation or cerebrospinal fistula. To the authors’ knowledge, there has been no previous study investigating the efficacy of this technique.

## Technical report

We performed a retrospective review of a prospectively collected database of patients that underwent a suboccipital craniotomy or craniectomy preserving the myofascial cuff compared to the traditional method. Each procedure had a midline incision, suboccipital craniotomy or craniectomy, dural graft, and sealant. This limited the major technical difference between techniques to the preservation of the myofascial cuff. We analyzed each patient’s postoperative course for any surgical complications, with particular attention paid to pseudomeningocele formation, CSF leak, and wound dehiscence, or meningitis. A pseudomeningocele was defined as a subcutaneous collection of cerebrospinal fluid palpable clinically and confirmed on imaging. A CSF leak was defined as a CSF-cutaneous fistula manifested by CSF leaking through the wound. All patients underwent postoperative imaging in the form of computed tomography (CT) or magnetic resonance imaging (MRI) immediately after surgery and at three to six months follow-up. All patients underwent regular postoperative visits at 14 days, six weeks, and three months. Patients with preoperative hydrocephalus, cerebellar hematoma or infarction requiring emergent surgery, or didn’t have all technical components performed were excluded.

### Surgical technique

**Figure 1:**
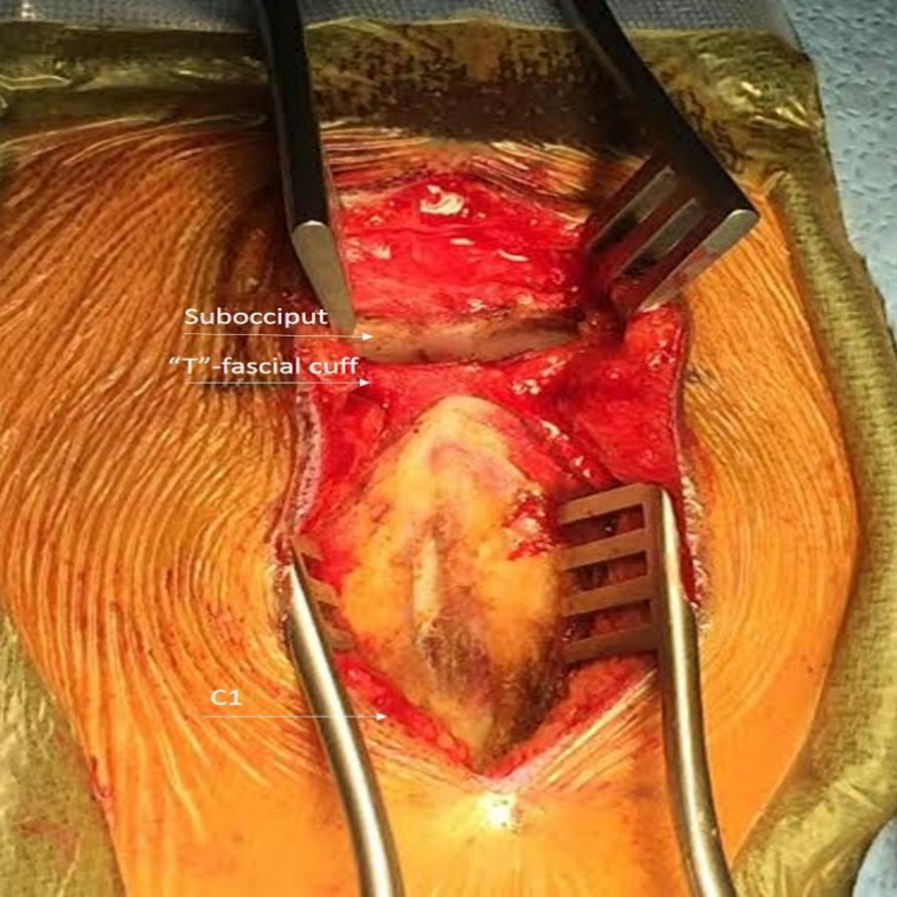
Intraoperative Photograph Highlighting the “T”-fascial Cuff During the Initial Exposure for a Suboccipital Craniectomy

The technique of suboccipital craniotomy or craniectomy has been previously described. A brief summary follows. Informed consent was obtained prior to all operations. After the induction of general anesthesia and appropriate intravenous access and neuromonitoring is obtained, the patient is then positioned prone in the Mayfield head holder in a flexed position to enhance exposure to the posterior fossa. A midline incision is planned from the inion or above to the level of the spinous process of C2. The incision is then carried deeper with Bovie® electrocautery (Bovie Med Corp, Clearwater, FL) in the midline to the level of the fascia and suboccipital muscles. The occipital bone at the superior nuchal line is invested with a tough fibrous band consisting of the attachments of the trapezius muscles bilaterally and deeper, the semispinalis capitis muscles. In the standard approach, all muscles are detached in the midline in a subperiosteal fashion from the inion down for deeper exposure of the bony elements. At this stage, rather than a wide detachment of the musculature, a “T” shaped incision is made of the fascia to leave a transverse fascial cuff at the superior nuchal line as is illustrated in Figure 1.

**Figure 2:**
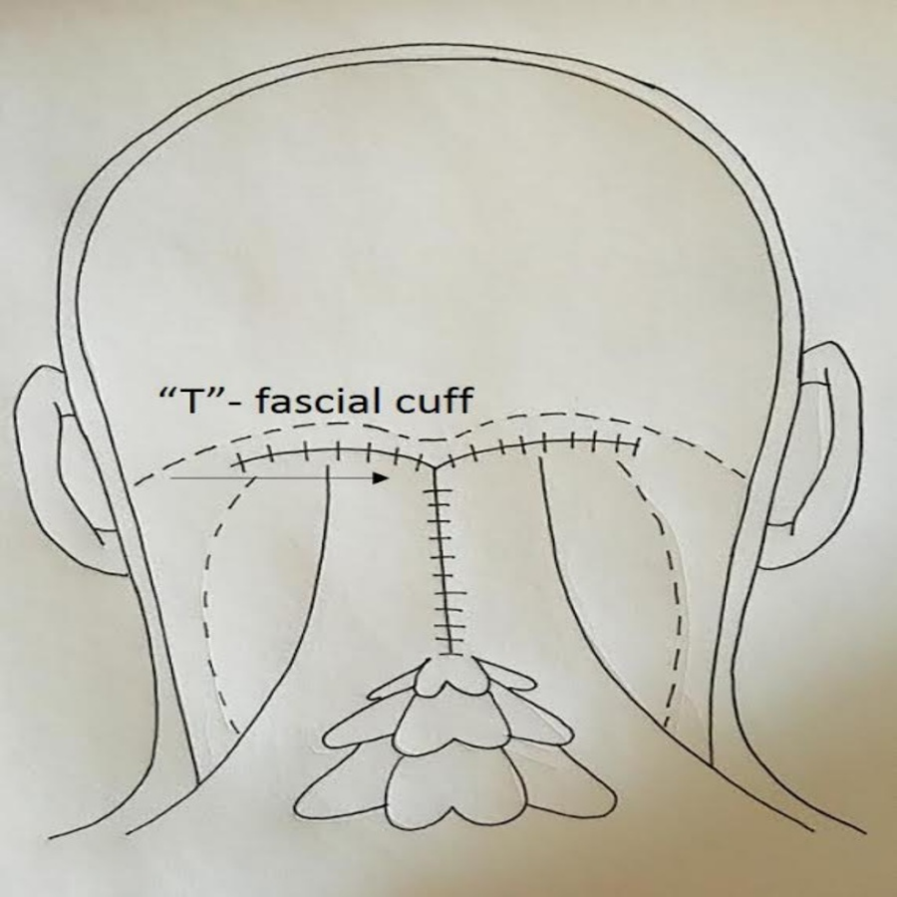
Artist’s Impression of the “T”-fascial Cuff During the Operative Approach for a Suboccipital Operation

The fascia on each side of the midline is then reflected laterally with the underlying musculature. After completion of the intended intradural portion of the operation, the dura is closed in a watertight fashion, when possible. A dural sealant is applied to the suture line. The muscles are reapproximated to reduce dead space with O Vicryl® sutures (Ethicon, New Jersey, USA). The fascia on each of the midline is then closed medially and superiorly along the transverse fascial cuff in watertight fashion with interrupted O Vicryl sutures as shown in Figure 2.   

The rest of the wound is then closed in layers in the usual manner followed by a non-absorbable suture used to close the cutaneous layer in a running fashion. A drain is not placed.

### Case illustration

**Figure 3:**
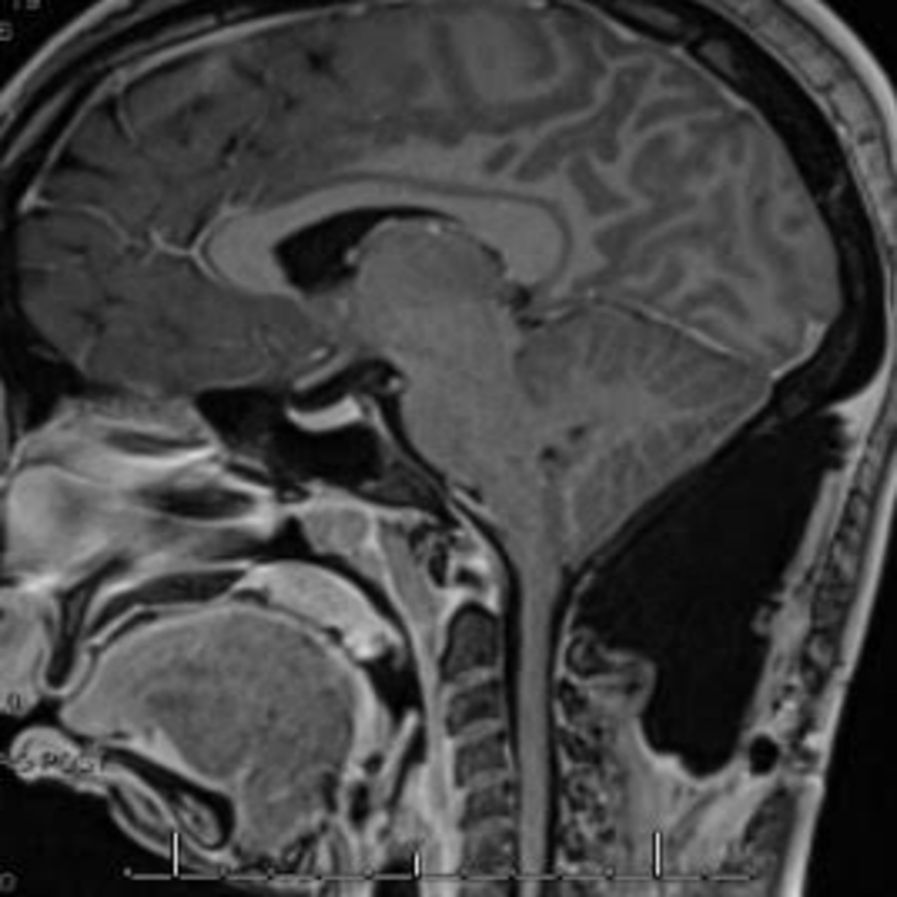
Postoperative Sagittal MRI After a Suboccipital Craniectomy in a Patient with a Delayed Diagnosis of Idiopathic Intracranial Hypertension

A 34-year-old non-obese female with occipital headaches diagnosed with a Chiari malformation underwent an uneventful suboccipital craniectomy with duraplasty. Due to worsening headaches, a postoperative MRI shown in Figure 3 confirmed a clinically relevant pseudomeningocele. A lumbar tap was performed to rule out meningitis.  

The opening pressure was noted to be above 50 cm H20 with remarkable relief of symptoms after the procedure. An ophthalmological evaluation confirmed papilledema. The patient was diagnosed with idiopathic intracranial hypertension (IIH) and was treated with a lumboperitoneal shunt. Her symptoms immediately improved and her pseudomeningocele resolved at six months. This case highlights the reliability of the “T”-fascial closure, which the authors believe helped prevent a CSF fistula despite intracranial hypertension.

### Results

Our retrospective review identified 33 patients matching the inclusion criteria. The mean age was 43 years old (range: 21-78). Twenty-one patients underwent surgery for a Chiari 1 malformation and 12 for primary or metastatic tumor. An autologous pericranium graft (n = 5) or bovine pericardial graft (n = 28) was performed in cases to achieve a watertight closure. In patients without a watertight closure, an onlay collagen-based graft was placed on top of the remaining dura. A dural sealant was used in all cases. The bone flap was repositioned anatomically in eight cases. All patients had a minimum of at least six months follow-up.

Overall, our cohort had a 21% (7/33) rate of clinical and radiographic pseudomeningocele formation with 9% (3/33) requiring surgical revision or a separate procedure. Of those utilizing the myofascial cuff preservation technique, there was a 12% (2/17) rate of a clinical and radiographic pseudomeningocele formation compared to 31% (5/16) in the standard cohort. The rate of permanent CSF diversion or revision was also less in the myofascial cuff preservation group at 6% (1/17) compared to 13% (2/16) in the standard. In all patients with persistent pseudomeningocele formation, there was no wound compromise nor evidence of CSF drainage.

## Discussion

Pseudomeningocele formation and CSF leak represent a significant complication after posterior fossa surgery. Cerebrospinal fluid leak rates in the published literature range between 1.5% - 23% [8-9]. A study by Dubey and colleagues revealed CSF leak, meningitis, and wound infection rates of 13%, 9.2%, and 7%, respectively, out of 500 patients. Reported pseudomeningocele rates from posterior fossa interventions range from 4% to 28% [10]. Litvack, et al. reported a 19.2% rate of pseudomeningocele formation after suboccipital decompression with duraplasty for Chiari I malformations. CSF-related complications can require readmissions and additional interventions with escalating costs.  

A variety of technical modifications has been proposed to reduce the rate of pseudomeningocele formation and CSF leak after posterior fossa surgery. Performing a craniotomy versus craniectomy reduces pseudomeningocele formation [11-12]. The use of autologous dural grafts as compared to non-autologous dural grafts can lower rates of wound complications [13]. Furthermore, a reduced rate of CSF-related complications can be seen with a variety of commercially available dural sealants [14-16].  

The technique described herein focuses on eliminating potential dead space and adding another water-tight layer to posterior fossa wound closures [17-18]. To our knowledge, this is the first closure technique of its kind to be analyzed as a method to decrease postoperative pseudomeningocele formation, although it has been postulated previously. These concepts have been applied to various surgical interventions, such as transsphenoidal surgery or skull base surgery, and highlight the importance of decreasing the potential space incited by the approach with an appropriate reconstruction of the operative defect [19].

The surgical technique is simple, fast, and only requires one additional step during exposure. Adding a second water-tight layer of closure is particularly helpful in patients with prior surgery or radiation where dural closure is not possible. In particular, the most superior aspect tends to be the most difficult position to close, due to the lack of attachment along the superior nuchal line. This is easily remedied by performing a T-shaped cuff at the initial dissection and allows for a firm attachment upon closure. The increased strength of this technique was demonstrated in the patient wherein a clinically relevant pseudomeningocele had formed but there was no drainage despite increased opening CSF pressures from the patient's idiopathic intracranial hypertension. This may be due to the fascial closure’s ability to allow for the more superficial layers to heal due to the impeded flow of CSF.

There are several inherent weaknesses of this analysis. First, because of the non-blinded retrospective nature of the study, there may be bias in reviewing the clinical and radiographic evidence of pseudomeningocele formation. Second, there is a multitude of factors that contribute to CSF-related complications after posterior fossa surgery. Our limited series cannot control for all these factors. The type of dural graft or dural sealant used was not standardized in all cases. A prospective randomized controlled study is needed to clearly demonstrate a reduced rate of CSF-related complications.

## Conclusions

Preservation of the myofascial cuff for watertight fascial closure during posterior fossa surgery reduces the rate of pseudomeningocele formation and cerebrospinal fluid leak as compared with standard techniques. This restorative anatomical technique is simple, easily adoptable without added morbidity, and worth incorporating. A larger prospective multi-institutional study is necessary to corroborate these findings.
